# Drivers and barriers to engaging with academia: a minority-ethnic medical student perspective

**DOI:** 10.1177/01410768211029156

**Published:** 2021-07-01

**Authors:** Carlos Curtis-Lopez, Daniel Robinson, Manasi Shirke, Catherine Dominic, Rakesh Patel

**Affiliations:** 1 School of Medical Sciences, University of Manchester, Manchester, M13 9PL; 2 School of Medicine, Queen’s University Belfast, Belfast, BT9 7BL; 3 Bart’s and the London School of Medicine, Queen Mary University of London, London, E1 2AD; 4 School of Medicine, University of Nottingham, Nottingham, NG7 2RD

## Introduction

The General Medical Council emphasises that ‘*newly qualified doctors must be able to apply scientific methods and approaches to medical research*’.^1^ However, opportunities for undertaking research during undergraduate training are limited, particularly for minority-ethnic students. Even before the limited number of opportunities are considered, there can be a number of barriers that may contribute to minority-ethnic students accessing opportunities to engage with research: (1) differential attainment and unconscious bias; (2) lack of role models from minority-ethnic backgrounds; and (3) difficulties forming meaningful mentor–mentee relationships. Each barrier contributes to individuals either not entering academia in the first place or attrition from academia as a consequence of the ‘leaky pipeline’ phenomenon.^2,3^ Given diversity of thinking and representation are associated with greater scientific impact and growth, not having minority-ethnic students consider academia is a lost opportunity for all.^4^ Through the lens of equality, diversity and inclusivity and prompted by recent events in the US, this issue is now being viewed differently, especially by medical students in the UK. Specifically, medical students currently entering university have a greater awareness around social justice and structural inequalities which may not be apparent to those already in the system. In this perspective, medical students at the start of our training share both evidence and reflections about barriers, and their experience of some of these during their time at university in relation to identifying possible drivers that may actually engage minority-ethnic students with academia. Although these medical student reflections are drawn from different UK universities, the lived experiences are likely to resonate with individuals from all minority-ethnic backgrounds where opportunities to engage with academia are not always equally accessible to all.

## Differential attainment and unconscious bias

A report produced by the Higher Education Statistics Agency identified that only 3% of the 15,560 first year UK PhD students identified as Black.^5^ UK Research and Innovation data also identified that only 30 (0.002%) from a total of 19,868 PhD studentships collectively granted by research councils from 2016 to 2019 were awarded to students who identified as Black Caribbean.^6,7^ These statistics are examples of the differential attainment or awarding gap between individuals from minority-ethnic backgrounds and those who identify as White. This phenomenon in terms of differences in achievement outcomes between groups is well described in medical education, medicine and across society.^8^ With respect to engaging minority-ethnic students into academia, even the perception that differential attainment exists around selection into academic training, award of funding or fellowships, or appointments into tenured posts may hinder medical students getting attracted into pursuing academia. While the discussion around race and unconscious bias within a medicine and medical education context is now maturing,^9^ not having a similar conversation within academic medicine in the broadest sense may prevent this barrier from being overcome.

## Role-models

A systematic review investigating medical students’ choices of sub-specialty identified 65% of respondents significantly considered advice from medical teachers or others in their career decision-making.^10^ Without role models from minority-ethnic backgrounds, there is every chance students may perceive a career in academia as not for them. In the literal sense, role models are individuals whose behaviours are imitated by others; therefore, having individuals in senior positions that students can see as either successes or someone they would like to be like is essential. Marian Wright Edelman, the American activist, coined the phrase ‘*you cannot be what you cannot see*’ and this statement particularly holds true for attracting minority-ethnic students at medical school into academia. Furthermore, medical schools having academics from minority-ethnic backgrounds in senior leadership roles is one thing; however, medical schools actively championing their successes is another. Progress has been made to increase the number of academics from minority-ethnic backgrounds,^11^ but not necessarily the same progress has been made celebrating them as people or their contributions to science, especially given the inspirational impact individuals such as Mary Seacole or Charles Richard Drew have had on future generations of students.

## Relationship building

A lack of mentoring was reported by 98% of participants as the most important barrier hindering career progression in academic medicine.^12^ For minority-ethnic students in particular, making, building and sustaining relationships are a challenge in a medical education context.^13^ Relationships in an academic context are complex for various reasons; however, for minority-ethnic students, cross-cultural differences influence the way relationships are experienced. In particular, these differences appear to lead to feelings of isolation,^13^ which can be particularly damaging in academia when collaborating with others is critical for success. There is also evidence that the quality of relationships can affect which opportunities may or may not be presented to students in the first place. Therefore, creating mentee–mentor relationships where individuals from minority-ethnic backgrounds can not only produce the outputs to survive in research, but also flourish and thrive in academia more broadly, is fundamental.^14^

## Impact on minority-ethnic students

In reviewing the evidence and reflecting over the barriers above, we look with apprehension at the scale of the challenges facing minority-ethnic students and doctors pursuing a career in academia. In particular, the evidence is stark and does not inspire confidence in us, or our chances of accessing the formal pathways into academia – the academic foundation programme, or a doctoral research fellowship. While individuals from minority-ethnic backgrounds in senior positions exist, they are both under presented and we cannot see their achievements celebrated by institutions, so drawing inspiration from their journeys in order to draw us into academia is also difficult. The problems around differential attainment and unconscious bias for us also triggers a feeling of apathy, as well as leading to a nagging thought at the back of our minds of ‘*why bother?*’ Furthermore, knowing the ‘*deck is stacked against us*’ from the outset in terms of building relationships with others, collaborative working, and experiencing the joy of discovery, makes it very difficult to know why minority-ethnic students like us should contemplate anything more than the conventional career pathway in medicine.

## Potential solutions and drivers to increasing engagement with academia

Like with most things in life, every problem can and should be seen as an opportunity. Nationally, major research funders and policymakers could start by making a real statement of intent addressing this problem, and develop an institutional strategy similar to one made by the Wellcome Trust for ensuring diversity of representation across their organisations.^15^ As part of a top-down approach, ensuring a proportion of those in senior leadership positions also reflect the entire population that they serve – be it patients and the public, or the medical student community – is important. Likewise, if those in senior leadership roles ensured there was diverse representation across the various committees, selection and award panels, more individuals from minority backgrounds could start to see themselves actually progressing in academia once they had entered. Finally, by having medical students from minority-ethnic backgrounds see something of themselves among those in senior leadership roles, or see more role models around them, it could be enough of a nudge to push them into having a conversation with academics in their institutions about pursuing a career in academia.

At a university level, data should be monitored around application, selection and progression of minority-ethnic students either in research training or other areas where they engage with research to ensure any differential outcomes are addressed. With this specific issue, trust comes from openness and transparency, and therefore, medical students seeing universities being prepared to act on any findings from data analysis is also important. Medical schools could re-double efforts within their universities around developing pathways for minority ethnic students to engage with research across undergraduate training. As well as creating positive learning environments during teaching activities, extending a sense of inclusivity into other spaces where minority-ethnic students engage with research or academia such as intercalated degrees, summer research internships and research electives is also necessary. Ensuring opportunities are advertised in such a way that these individuals can get to see them, and by extension see themselves having a chance of getting them when applying is critical.

At a departmental level, academics should always be on the lookout to create opportunities for building relationships with the diverse and many medical students, as opposed to the privileged few. As part of a bottom-up approach, departments could lead on facilitating students connecting meaningfully with researchers and potential supervisors, so any barriers related to apprehension around talking with academic staff is minimised. Again, rather than offering a particular type of sponsorship for students from specific backgrounds to access opportunities through external professional organisations, investing in the infrastructure of existing mentoring schemes within medical schools or the wider university, including reverse mentoring initiatives, all students would again benefit, including those from minority-ethnic backgrounds. Finally, academics within departments alongside delivering their teaching, could also deliver simple lectures on how to publish, or engage with student medical societies to ensure research opportunities are known to many rather than the few. In these last few examples, such small and simple first steps could just be enough for drawing in all students into academia, but for those from the minority-ethnic backgrounds, it could be the significant gesture that made them believe they could be future researchers.

## Declarations

**Ethics approval:** Not applicable.

**Guarantor:** Guarantor is Rakesh Patel.

**Contributorship:** CCL and RP contributed to the conceptualising of this perspective. CCL, DR, CD and MS conducted the review of the literature. CCL, DR, CD and MS wrote the first draft of this manuscript. CCL, DR, CD, MS and RP made critical revisions of this paper. All authors have approved the final manuscript and are willing to take responsibility for the content.
